# Increased Nitroxidative Stress Promotes Mitochondrial Dysfunction in Alcoholic and Nonalcoholic Fatty Liver Disease

**DOI:** 10.1155/2013/781050

**Published:** 2013-04-03

**Authors:** Byoung-Joon Song, Mohamed A. Abdelmegeed, Lauren E. Henderson, Seong-Ho Yoo, Jie Wan, Vishnudutt Purohit, James P. Hardwick, Kwan-Hoon Moon

**Affiliations:** ^1^Section of Molecular Pharmacology and Toxicology, Laboratory of Membrane Biochemistry and Biophysics, National Institute on Alcohol Abuse and Alcoholism, 9000 Rockville Pike, Bethesda, MD 20892, USA; ^2^Department of Forensic Medicine, Seoul National University College of Medicine, Seoul 110, Republic of Korea; ^3^National Institute on Drug Abuse, Bethesda, MD 20892, USA; ^4^Department of Integrative Medical Sciences, Northeastern Ohio University College of Medicine, Rootstown, OH 44272, USA; ^5^Department of Molecular Pharmacology and Therapeutics, Loyola University Medical Center, Maywood, IL 60153, USA

## Abstract

Increased nitroxidative stress causes mitochondrial dysfunctions through oxidative modifications of mitochondrial DNA, lipids, and proteins. Persistent mitochondrial dysfunction sensitizes the target cells/organs to other pathological risk factors and thus ultimately contributes to the development of more severe disease states in alcoholic and nonalcoholic fatty liver disease. The incidences of nonalcoholic fatty liver disease continuously increase due to high prevalence of metabolic syndrome including hyperlipidemia, hypercholesterolemia, obesity, insulin resistance, and diabetes. Many mitochondrial proteins including the enzymes involved in fat oxidation and energy supply could be oxidatively modified (including *S*-nitrosylation/nitration) under increased nitroxidative stress and thus inactivated, leading to increased fat accumulation and ATP depletion. To demonstrate the underlying mechanism(s) of mitochondrial dysfunction, we employed a redox proteomics approach using biotin-*N*-maleimide (biotin-NM) as a sensitive biotin-switch probe to identify oxidized Cys residues of mitochondrial proteins in the experimental models of alcoholic and acute liver disease. The aims of this paper are to briefly describe the mechanisms, functional consequences, and detection methods of mitochondrial dysfunction. We also describe advantages and limitations of the Cys-targeted redox proteomics method with alternative approaches. Finally, we discuss various applications of this method in studying oxidatively modified mitochondrial proteins in extrahepatic tissues or different subcellular organelles and translational research.

## 1. Introduction

Mitochondria are responsible for the production of energy in the form of ATP which is used by every cell for its survival and function. In addition, mitochondria play a critical role in fatty acid oxidation, antioxidant defense, apoptosis, intermediary metabolism (including ammonia, urea, heme, steroid, pyrimidine, one carbon transfer, and glutamine metabolism), and so forth, [1–3]. The mitochondrial fat oxidation pathway is very important in providing alternative energy (e.g., ketone bodies) when glucose is supplied in limited amounts or not utilized for maximal energy production through the mitochondrial tricarboxylic acid cycle under various disease states [1]. It is known that heavy and chronic alcohol (ethanol) intake causes alcoholic fatty liver, steatohepatitis (inflammation), fibrosis, cirrhosis, and carcinogenesis in humans and experimental animal models [4, 5]. Because of the high solubility of alcohol, it is distributed in most tissues; therefore, excessive alcohol intake (e.g., binge or chronic heavy alcohol drinking) can damage virtually all tissues including liver, heart, brain, lung, pancreas, and testis [6–8]. Continuous consumption of calorie-enriched high-fat diets or administration of a choline-deficient diet in experimental animals can also cause significant fatty liver disease (i.e., nonalcoholic fatty liver disease) [9, 10], which are clinically similar to those of the aforementioned alcoholic fatty liver disease. In addition, acute and chronic infection from hepatitis viruses can increase oxidative stress and cause various liver diseases including fibrosis and cirrhosis depending on the degree of host-viral interactions [11]. Certain drugs such as the antibreast cancer agent tamoxifen and antiretroviral drugs including AZT (zidovudine) can promote fatty liver disease ([12], and references herein). Likewise, abused substances such as marijuana (cannabinoids), nicotine (a major component of tobacco smoke), and 3,4-methylenedioxymethamphetamine (MDMA, ecstasy) can lead to hepatic steatosis and inflammation (steatohepatitis) [13–15] as well as tissue injury in extrahepatic organs including brain and heart [16–19].

**Figure 1:**
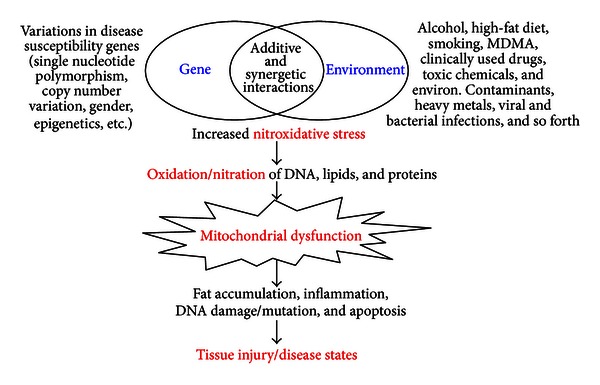
Synergistic interaction between gene and environment. Many toxic agents alone or in combination with other comorbidity factors including genetic elements synergistically interact and produce ROS/RNS, which decrease the levels of antioxidants and inhibit protective defensive enzymes, resulting in increased nitroxidative stress. Consequently, mitochondrial DNA, lipids, and proteins are oxidized and/or nitrated, leading to mitochondrial dysfunction, accompanied with fat accumulation, inflammation, ATP depletion, necrosis/apoptosis, and DNA damage. All these changes likely contribute to tissue injury, as observed in many disease states.

Regardless of the etiological factors, both alcoholic and nonalcoholic fatty liver diseases can result from impaired mitochondrial functions (i.e., mitochondrial dysfunction) with suppressed *β*-oxidation of fatty acids [2, 12, 20]. Furthermore, some exogenous agents (e.g., alcohol or high-fat diet) alone or in combination with other gene-related comorbidity risk factors can damage the liver in a synergistic/additive manner to rapidly promote or worsen the preexisting conditions, as observed in rats [21]. It is also known that other extrahepatic tissues can be negatively affected or damaged by the combination of environmental factors (e.g., excessive amounts of alcohol consumption, smoking, drugs, and abused substances) and genetic factors (e.g., variations in disease susceptible genes such as a dominant negative mutation of mitochondrial aldehyde dehydrogenase (ALDH2) gene, frequently found in many East Asians [22–25], diabetes, obesity, and neurodegenerative diseases) (Figure 1). In fact, mitochondrial dysfunction could serve as a major contributor in many disease states such as alcohol- or drug-mediated tissue injury, aging, cancer, diabetes, and various neurodegenerative diseases [20, 26–31], even though the etiological factor for each disease state is different. Despite the well-established role of mitochondrial dysfunction in many disease states, it is poorly understood how mitochondrial dysfunction occurs in these pathological conditions. In this paper, we briefly describe the role of nitroxidative stress in promoting mitochondrial dysfunction and its pathophysiological consequences. We also describe the detection of oxidatively modified mitochondrial proteins in experimental models of alcoholic and nonalcoholic fatty liver disease with redox-based proteomics approaches. Finally, we discuss potential limitations and applications of redox proteomics approaches in studying oxidatively modified proteins in different subcellular fractions in various tissues as well as future translational research.

## 2. Role of Oxidative Stress in Promoting Mitochondrial Dysfunction

Under normal conditions, approximately 1%-2% of oxygen leaks out as reactive oxygen species (ROS) from the mitochondrial electron transport chain (ETC) [32]. These amounts of ROS, adequately handled by the cellular defense systems under normal conditions, can regulate various cellular signaling pathways, as recently discussed [33]. However, under pathological conditions or after exposure to certain toxic agents including large quantities of alcohol, abused substances, or other therapeutic drugs [17–20, 30], greater amounts of ROS are leaked from the mitochondrial ETC, possibly at the sites of Complex I (NADH ubiquinone oxidoreductase) and Complex III (ubiquinone cytochrome c oxidoreductase), as shown in alcohol-exposed hepatocytes [34]. Ironically, mitochondria, a major source of cellular ROS, become a main target of oxidative damage because of the relatively low levels of antioxidants, such as reduced glutathione (GSH), in mitochondria compared to cytosol [35]. Consistent with a notion of ROS-mediated damage, mitochondria from tissues in animal disease models and/or human diseases show abnormal and irregular shapes and decreased functions [36, 37].

Besides the ROS generation from the mitochondrial ETC, other cellular enzymes are also known to produce ROS and reactive nitrogen species (RNS) including nitric oxide (NO). These enzymes include NADPH oxidase and myeloperoxidase in phagocytic immune cells, ethanol-inducible cytochrome P450 2E1 (CYP2E1) and CYP4A isozymes in endoplasmic reticulum (ER), cytosolic xanthine oxidase, and nitric oxide synthase isozymes including the inducible form (iNOS) in activated Kupffer cells and/or recruited neutrophils [38–45]. Many of these prooxidant enzymes are induced or activated after exposure to potentially toxic agents such as alcohol, MDMA, and high-fat diets. Elevated ROS leaked from the mitochondrial ETC and produced by these enzymes lead to increased production of a potently toxic peroxynitrite (ONOO^−^) in the presence of NO. Peroxynitrite can covalently modify various proteins through nitration of Tyr residues [46, 47] and *S*-nitrosylation of Cys residues [48]. In fact, elevated ROS/RNS under pathological conditions suppress the activities of various antioxidant enzymes including mitochondrial superoxide dismutase (SOD2), catalase, glutathione peroxidase, and glutathione reductase while they can also decrease the levels of cellular antioxidants such as GSH and vitamins, causing increased nitroxidative stress.

Under the conditions of elevated nitroxidative stress, mitochondrial DNA, proteins, and lipids become covalently modified by oxidation, nitrosation, and/or nitration. Increased nitrosative stress can lead to various reactions, including *N*-, *O*-, and *S*-nitrosations, that modify structure of various proteins, as reviewed [49–52]. Nitrative stress represents a condition where excessive ROS reacts with NO to produce potently toxic peroxynitrite, which can nitrate Tyr residues of various proteins to produce 3-nitroTyr, frequently used as a stable marker for nitrative stress [48–52]. These types of modifications of cellular macromolecules likely contribute to alteration of their normal functions [53–55]. Deletion and/or mutation through oxidative modifications of mitochondrial DNA are particularly important, since they encode 13 polypeptides, all of which are subunits of the 4 mitochondrial ETC proteins (i.e., Complexes I, III, IV, and V) [30, 31]. Mitochondrial DNA and proteins are more susceptible to oxidative/nitrative damage due to the absence of protective antioxidant protein catalase, histones, or polyamines and a relatively low activity of DNA repair enzyme in mitochondria compared to nuclei ([56], and references herein). One report suggested that the rate of mutation in mitochondrial DNA is 10-fold higher than that in the nuclear DNA [29]. In addition, the level of mitochondrial GSH is relatively low compared to that in cytosol because it has to be imported into mitochondria through the specific GSH transporter protein due to the absence of its synthesis in mitochondria. Furthermore, chronic alcohol exposure impairs the GSH transporter protein, leading to a selective deficiency of GSH in mitochondria ([35], and references herein). It is thus reasonable to assume that oxidative damage and/or deletion of mitochondrial DNA [57, 58] may lead to reduced expression and function of mitochondrial ETC proteins, contributing to greater ROS production, as shown in alcohol-exposed rats [59].

Oxidation of lipids also produces potently cytotoxic lipid peroxides such as 4-hydroxynonenal (4-HNE) and malondialdehyde (MDA). These lipid peroxides can suppress the activities of many mitochondrial proteins such as ALDH2 [60], involved in the metabolism of reactive acetaldehyde and 4-HNE, and Sirt3, NAD^+^-dependent deacetylase [61], through covalent modifications (mostly via adduct formation with many amino acid residues including Cys, His, and Lys) [54, 55]. These lipid peroxides can alter the cell membrane functions and promote fibrosis through activation of stellate cells with elevated production of collagen and proinflammatory cytokines/chemokines that lead to recruitment of neutrophils and activation of macrophage Kupffer cells. All these events contribute to profound mitochondrial dysfunction with increased fat accumulation and tissue injury in liver and various extrahepatic organs [4, 5, 28, 29].

Numerous investigators have reported the mechanisms and consequences of increased levels of oxidized mitochondrial DNA and lipid peroxides in various human disease states as well as experimental models for human disease [12, 53, 57, 62, 63]. Compared with the numerous reports about oxidative modifications of (mitochondrial) DNA and lipids, a much smaller number of reports systematically dealt with the oxidatively modified (mitochondrial) proteins under increased nitroxidative stress associated with many disease states. We believe that part of the reason for the relatively fewer reports on protein oxidation, nitrosation, and nitration might be due to the requirement for specific reagents, the lack of suitable methods to systematically identify and purify oxidatively modified proteins, and the relatively late development of highly sensitive mass spectral instruments. Because of the delayed development of specific methods to systematically study oxidatively modified proteins, the following questions have been poorly answered: (1) do we have a suitable method to systematically identify the oxidatively modified proteins and study the causes of mitochondrial dysfunction compared to global analysis of the changes in mitochondrial proteins? (2) what are the sources of ROS/RNS in enhancing oxidative protein modifications (including oxidation and nitration)? (3) which (mitochondrial) proteins are oxidatively modified? (4) are their activities/functions altered following oxidative modifications? (5) what are the functional implications of oxidized proteins in mitochondrial dysfunction and certain disease states such as various models of fatty liver disease? and (6) can the oxidative protein modifications and subsequent mitochondrial dysfunction be prevented with a potential therapeutic agent? and (7) how do current treatment modalities of fatty liver disease impact the function and oxidation of mitochondria proteins?

## 3. Consequences of Mitochondrial Dysfunction

Although both genetic and environmental factors synergistically promote mitochondrial dysfunction in various pathophysiological conditions, increased nitroxidative stress represents one important common factor (Figure 1). Despite the well-established pathological role of elevated nitroxidative stress, it has been poorly understood which mitochondrial proteins are oxidatively modified and whether their functional alterations cause mitochondrial dysfunction prior to full-blown tissue damage determined by histological and biochemical assessments. Therefore, we hypothesized that mitochondrial dysfunction is mediated by covalent modifications (e.g., oxidation, nitrosation, nitration, phosphorylation, acetylation, etc.) of various mitochondrial proteins, leading to their inactivation or loss of their biological functions. In case of mitochondrial dysfunction, we expect to observe increased levels of energy depletion, lipid peroxidation, and fat accumulation possibly due to suppressed activities of the enzymes involved in ATP synthesis, ALDH2-mediated metabolism, and fatty acid *β*-oxidation, respectively. In this paper, we briefly describe the functional changes of oxidatively modified ATP synthase, ALDH2, and 3-ketoacyl-CoA thiolase (thiolase) as examples.

Cederbaum et al. [64] and Chen et al. [65, 66] reported that alcohol administration directly or indirectly suppressed the activities of mitochondrial Complex enzymes through increased oxidative stress. It is also likely that decreased amounts of Complex I (NADH ubiquinone oxidoreductase), and Complex IV (cytochrome c oxidase) observed in alcohol-exposed rats [59] could have contributed to suppression of their catalytic activities. Inhibition of these Complex activities could cause more ROS leakage from the mitochondrial ETC, as observed in alcohol-exposed hepatocytes [34]. In addition, ROS can be produced at the site of mitochondrial Complex II (succinate dehydrogenase) [33].

By performing redox proteomics analysis, we sought to identify oxidatively modified mitochondrial Complex proteins in rat livers exposed to chronic or binge alcohol compared to control rats [67]. Oxidation of many mitochondrial proteins including Complexes I, III, and V protein subunits was detected. Activity measurement showed that ATP synthase (Complex V) was significantly inhibited in alcohol-exposed rats possibly through oxidative modifications (including nitration) of the enzyme [67]. Immunoblot analysis with the anti-3-nitroTyr antibody verified the presence of a 3-nitroTyr-reactive band in the immunoprecipitated ATP synthase protein only from alcohol-exposed livers, suggesting its nitration. Mass spectral analysis confirmed the nitration of Tyr residues in the catalytic *β* subunit, which does not have a Cys residue but must have been copurified with the Cys-containing *α* subunit of ATP synthase [67]. Nitration of the catalytic subunit of ATP synthase could have contributed to its inactivation. The markedly suppressed ATP synthase likely resulted in significantly decreased ATP levels, as observed in many pathological conditions [68, 69].

Systematic redox proteomics analysis of oxidatively modified mitochondrial proteins in experimental models of acute liver disease caused by alcohol, MDMA, or hepatic I/R injury revealed the detection of all 4 enzymes (medium-chain fatty acyl-CoA dehydrogenase, enoyl-CoA hydratase, 3-hydroxyacyl-CoA dehydrogenase, and thiolase), involved in the mitochondrial *β*-oxidation of fatty acids [15, 67, 70]. Activity measurement of thiolase, the last enzyme in the mitochondrial *β*-oxidation pathway of fatty acids, showed inhibition of this enzyme in the animal models of alcoholic fatty liver disease, possibly through oxidative modification of the active site Cys residues (Cys^92^ and Cys^382^) of thiolase [67]. It is likely that the active site Cys residues can undergo oxidation including *S*-nitrosylation as well as formation of adducts with 4-HNE or MDA, since their levels can be increased through lipid peroxidation under oxidative stress. Because of the oxidative modifications of 3 other enzymes in the *β*-oxidation pathway, as mentioned above, we expect that their activities could be altered in alcohol-exposed rats. In addition, it is likely that the activity of the NAD^+^-dependent 3-hydroxyacyl-CoA dehydrogenase could be compromised due to significant changes in the NAD^+^/NADH levels following alcohol intake [4, 39]. At any rate, oxidative modifications and subsequent inhibition of at least one of the mitochondrial fat oxidation pathway enzymes correlated with hepatic inflammation and fat accumulation, as assessed by biochemical measurements of triglycerides as well as histological evaluations [67].

From the redox proteomics analysis, we also identified a few oxidatively modified mitochondrial ALDH isozymes in the mitochondria from alcohol-exposed rats [67]. The ALDH gene family [71, 72] represents a large number of NAD(P)^+^-dependent dehydrogenases (defensive enzymes) [73–75] involved in the cellular metabolism of reactive and cytotoxic aldehyde carbonyl compounds such as acetaldehyde, MDA, 4-HNE, and other lipid aldehydes that are produced during the lipid peroxidation process [54, 76]. The ALDH isozymes including mitochondrial ALDH2 are known to be inactivated by genetic/environmental factors [22–25, 71, 72, 77–79] and in many disease states [80–83]. Many isozymes of the *ALDH* gene family members including retinal aldehyde dehydrogenase (ALDH1A1/2/3) and 10-formyltetrahydrofolate dehydrogenase (ALDH1L1) contain the highly conserved active site Cys residue [84]. Oxidative modifications of the active site and other critical Cys residues of a cytosolic high-Km ALDH1A1 and mitochondrial low-Km ALDH2 (Km for acetaldehyde ≤ 0.2 *µ*M) [75], an important enzyme in the metabolism of toxic acetaldehyde produced during ethanol oxidation, contribute to inhibition of their activities [67, 84]. Under our experimental conditions, we did not observe any significant changes in the ALDH1A1 or ALDH2 protein content, strongly suggesting that inhibition of these ALDH enzymes could be due to covalent modifications of critical Cys residues by *S*-nitrosylation and other oxidative modifications, as discussed [85, 86]. Our results are consistent with those of Venkatraman et al. who reported inhibition of ALDH2 activity without change in its content in alcohol-exposed rats, although the suppressed ALDH2 activity was not restored after incubation with 0.3 mM *β*-mercaptoethanol, suggesting an irreversible ALDH2 inactivation in their model [87]. Although we have not measured the specific activities of other mitochondrial ALDH isozymes such as ALDH5A1 (NAD^+^-dependent succinic semialdehyde dehydrogenase involved in the catabolism of the neurotransmitter gamma-aminobutyric acid), ALDH6A1 (methylmalonate semialdehyde dehydrogenase), and ALDH7A1 (*α*-aminoadipic semialdehyde dehydrogenase) [71, 88], their activities are likely suppressed due to the highly conserved active site Cys residue by a similar mechanism, as recently demonstrated with mitochondrial ALDH2 [67, 70, 85] and cytosolic ALDH1A1 [84]. Because of the inactivation of ALDH1A1, ALDH2, and other ALDH isozymes through covalent modifications such as *S*-nitrosylation [67, 85], phosphorylation [89], and other modifications [86] including adduct formations with MDA or 4-HNE [60, 90] or reactive drug metabolites [78, 79], we expect increased levels of highly reactive and cytotoxic carbonyl compounds including acetaldehyde, 4-HNE, and MDA.

**Figure 2:**
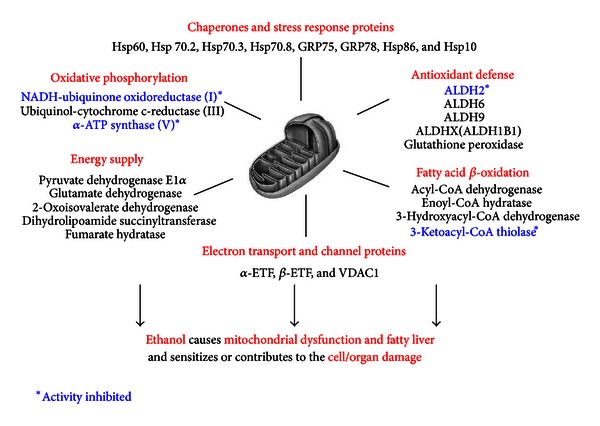
Summary of oxidatively modified mitochondrial proteins in alcohol-exposed rat livers. Oxidized mitochondrial proteins were purified from alcohol-exposed rats and dextrose-exposed pair-fed controls, identified by mass spectral analysis, and then grouped under different functions, as adapted from [67].

In addition to ALDH isozymes, many other mitochondrial proteins were oxidatively modified and inactivated in alcohol-exposed rat livers [67] (Figure 2). We also observed similar patterns of oxidatively modified mitochondrial proteins in the animal models of acute liver disease from hepatic I/R injury [70] or MDMA exposure [14, 91], or during fasting-related oxidative stress [92]. We expect that similar patterns of oxidative modifications of many mitochondrial proteins would be identified in experimental models of nonalcoholic fatty liver disease caused by high fat diets [76] or methionine/choline-deficient diets [10, 45], based on increased oxidative stress and similar courses of disease progress between alcoholic and nonalcoholic fatty liver diseases [9].

## 4. Mitochondrial Dysfunction and Fatty Liver Disease Studied with Redox Proteomics Approaches

It would be ideal if the activity change of each oxidatively modified mitochondrial protein can be measured to accurately assess the degree and functional implications of mitochondrial dysfunction in different pathological conditions or before and after exposure to a potentially toxic agent including alcohol or other abused substances such as MDMA and nicotine. However, this task may be impractical due to a relatively small yield of mitochondrial proteins from whole tissue extracts and the requirement for a large amount of mitochondrial proteins for direct activity measurement. As alternative approaches, other methods including global gene expression DNA microarrays and proteomics analyses have been used to indirectly assess the relative degree of mitochondrial dysfunction [68, 93]. Although DNA microarrays allow global analysis of gene expression changes in disease states versus presumably normal control conditions, the results do not always accurately reflect the changes in the final protein amounts or activities of (mitochondrial) proteins [68, 94, 95]. Therefore, a few proteomics methods with or without gel-based tools have recently been developed to quantitatively evaluate the changes in the expressed levels of many proteins in two different specimens (e.g., disease states and apparently normal controls). The recent methods include fluorescence 2D difference in gel electrophoresis (2D DIGE) [96], cleavable isotope-coded affinity tag (cICAT) [97], isobaric tags for relative and absolute quantitation (iTRAQ) [98], multidimensional protein identification technology (MudPIT) [99], and so forth. These global proteomics methods can also be used to investigate the quantitative changes in mitochondrial proteins. In addition, a conventional proteomics approach consisting of comparative 2D gel analysis was successfully used for analyzing the protein changes in the whole tissue extracts [83, 100] or the enriched mitochondrial fractions from alcohol-exposed rats compared to corresponding controls [59]. However, despite the advantages and merits, all these global proteomics approaches may not necessarily provide valuable information about functional changes of the target proteins, since the activities of many (mitochondrial) proteins can be suppressed without significant quantitative changes of their contents, as demonstrated with ALDH2 [67, 70, 87]. These results rather suggest that the functions/activities of many (mitochondrial) proteins could be altered through posttranslational modifications such as oxidation of many amino acids including Cys residues, nitration of Tyr residues, phosphorylation of Ser, Thr and Tyr residues, acetylation of Lys residues, glycosylation of Asn residues, and adducts formation with 4-HNE, or reactive drug metabolites [65–67, 77–79, 86, 101–103]. Instead of studying covalent modifications of various amino acid residues (e.g., Cys, Trp, His, Met, Pro, Tyr, and Lys) that can be differently modified [55], we and other scientists focused on targeted proteomics approaches aiming for oxidatively modified Cys residues of many proteins that can be purified by affinity matrix, displayed on 2D PAGE gels, stained with silver, and identified by mass-spectral analysis. Identification of the oxidatively modified proteins detected with the Cys-targeted redox proteomics approach and a literature search for active site Cys residues of each oxidized protein allow us to predict functional changes (e.g., potential inhibition) of the oxidized proteins/enzymes even in the absence of any changes in protein content.

In order to study oxidative inactivation of many mitochondrial proteins responsible for causing mitochondrial dysfunction, a few redox proteomics approaches have been used to identify oxidized Cys residue(s) of many proteins by using ICAT [97], biotin-labeled iodoacetamide (BIAM) [104], 4-iodobutyltriphenyl-phosphonium [87], or biotin-*N*-maleimide (biotin-NM) [105], being used as a major sulfhydryl-detecting agent in each approach. Although each detection method has its own merit, our approach using biotin-NM as a specific probe for oxidized Cys residues revealed a positive correlation between the levels of oxidative stress in pathological conditions and the number of oxidized proteins [105]. Therefore, we believe that the redox proteomics method using biotin-NM as a sensitive probe for detecting oxidized Cys residues may have a significant advantage over the other redox proteomics methods, where the number of oxidized proteins could be inversely correlated with the increased oxidative stress and oxidized Cys residues do not efficiently react with iodoacetamide-based sulfhydryl reagents BIAM and ICAT [87, 97, 104]. The specific procedures, advantages, limitations, and alternative approaches of our method and its comparison with other redox proteomics methods have been recently described in detail [106]. Excellent review articles on the theories, benefits, and limitations of various redox proteomics approaches from other laboratories are also available [107–112].

**Figure 3:**
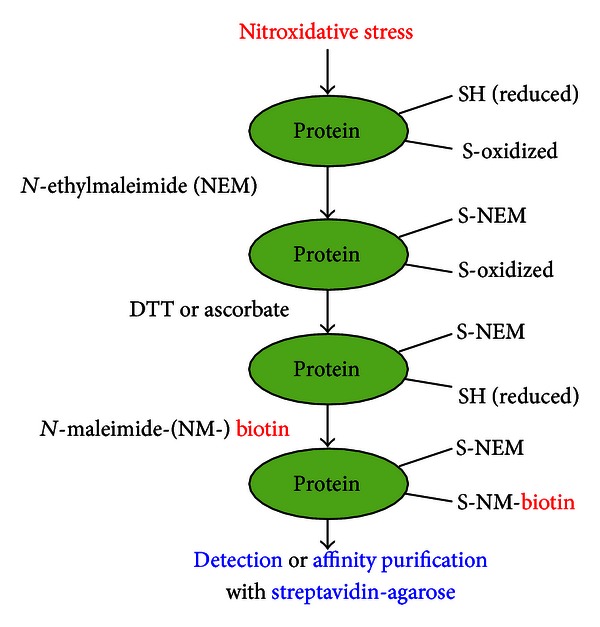
A schematic diagram describing a redox proteomics approach using biotin-NM as a specific probe for detecting or purifying oxidatively modified proteins, as adapted from [106].

To avoid redundancy with some of the previous reviews [106–112], we briefly describe the procedure of our simple redox proteomics method as outlined in Figure 3. Many sulfhydryl groups of various proteins can be oxidatively modified (e.g., sulfenic acid, disulfide, sulfinic acid, sulfonic acid, and mixed disulfides including *S*-nitrosylation) under increased nitroxidative states. The remaining free Cys thiols of various proteins are initially reacted with *N*-ethylmaleimide (NEM), which irreversibly blocks the free thiols. After removing excess NEM by the first gel filtration step, the oxidized Cys residues including mixed disulfides are reduced to free Cys thiols with DTT. The newly reduced free Cys thiols are then switched with biotin-NM. After removing excess biotin-NM with the second gel filtration step, biotin-labeled oxidized proteins are detected by immunoblot analysis or affinity-purified with streptavidin-agarose beads for further characterizations. After washing the nonspecifically bound proteins, agarose-bound biotin-NM-labeled oxidized proteins are dissolved and analyzed by 1-D PAGE for detection with anti-biotin monoclonal antibody or 2D PAGE for protein display followed by identification by mass spectrometric analysis. By using a mild reducing agent ascorbate or GSH [113, 114] instead of DTT in the reduction step of NEM-modified proteins, we can specifically detect proteins with mixed disulfides (e.g., *S*-cysteinylation, *S*-nitrosylation, *S*-glutathionylation, and *S*-succinylation), although the ascorbate-mediated biotin-switch method can produce false-positive artifacts [115, 116].

In fact, we used this redox proteomics approach to identify many oxidized mitochondrial proteins in ethanol-exposed human hepatoma E47-HepG2 cells with stably transfected human CYP2E1, which can produce ROS during ethanol metabolism [38, 39]. The number of oxidized proteins positively correlated with the ethanol concentration and ethanol exposure time as well as the presence of CYP2E1. By contrast, we observed a very limited number of oxidized mitochondrial proteins in control samples from E47-HepG2 cells that were not treated with ethanol or HepG2 cells without transfected CYP2E1 [105]. This biotin-switch redox proteomics approach was subsequently applied in analyzing oxidatively modified mitochondrial proteins in experimental animal models of alcoholic fatty liver [67] and other models of acute liver disease [15, 70]. The results from these studies revealed that many mitochondrial proteins were oxidatively modified and some of their activities we that measured were inhibited. Temporal analyses of oxidatively modified proteins and liver histology indicated that mitochondrial dysfunction takes place long before appearance of full-blown liver damage including necroinflammation [70]. Based on these results, oxidative modification and inactivation of many mitochondrial proteins cause mitochondrial dysfunction, which then contributes to tissue injury observed at later time points. Furthermore, we expect that this redox proteomics approach can be successfully used to identify and study functional alterations of oxidatively modified proteins in mitochondrial dysfunction and tissue injury in various organs of human disease specimens.

Although these redox proteomics approaches can be used as surrogate methods to estimate the degree of mitochondrial dysfunction, they do have a few limitations. For instance, we believe that the actual number of oxidized proteins could be much greater than we observed in our studies because of relatively poor sensitivities of gel-based redox-proteomics methods in detecting proteins expressed in low quantities. In fact, our systematic analysis using the Cys-targeted biotin-switch method was unable to detect oxidative modifications of DNA repair enzymes such as O^6^-methylguanine-DNA-methyltransferase, although this enzyme contains Cys in its active site and can be inactivated through *S*-nitrosylation [117]. By the same token, we also expect that some key enzymes involved in the cell metabolism/signaling pathways like mitochondrial Sirt3 [118–120] or certain transcription factors that contain critically important Cys residues could not be detected by the redox proteomics approaches although they could be oxidatively modified and thus inactivated by increased oxidative/nitrative stress under pathological conditions. In addition, some oxidized or nitrated proteins could be rapidly degraded through ubiquitin-dependent and -independent proteolysis [55, 121–124] and thus could not be detected by the current redox proteomics methods.

We believe that these limitations associated with various redox proteomics approaches can be overcome by functional analysis (including enzyme activity measurement) supplemented with immunoprecipitation of a target protein, albeit a low level of expression, followed by immunoblot analysis with anti-Cys-*S*-NO, anti-glutathione, and anti-3-nitroTyr antibody for detecting *S*-nitrosylation, *S*-glutathionylation, and nitration, respectively. A few examples of other critical proteins, that were expressed in small quantities and not detected by the systematic redox proteomics analyses but reported to be suppressed through oxidative modifications, were recently discussed [106]. It is of interest whether other critical mitochondrial proteins expressed in small quantities (e.g., sirtuin 3) can be inactivated through oxidative modifications, thus directly contributing to mitochondrial dysfunction with imbalanced energy supply [118], intolerance to cold exposure with decreased fat oxidation during fasting [119], and decreased mitochondrial complex activities [120], as observed in mice deficient of mitochondrial *sirtuin 3* gene. A recent report revealed that Cys^280^, a critical zinc binding residue, of Sirt3 is modified by 4-HNE, resulting in its allosteric inactivation [61]. It would also be of interest to study the potential mechanisms of oxidative inactivation or degradation of some transcription factors such as NFkB as observed in alcohol-exposed genetically obese mice [125] and PPAR*α*, a key regulator of the enzymes involved in the fat metabolism [126] and shown to be decreased in alcohol-fed mice [127], in mice with nonalcoholic steatohepatitis [128], or in acetaminophen-mediated acute liver damage [129]. Finally, the study of ER-associated drug metabolizing proteins such as cytochromes P450, that have Cys residues at their catalytic sites, may provide important insights in uncoupling of the catalytic cycle during adverse drug reactions [130].

Another limitation of the redox proteomics could be reasoned that Cys residues of many proteins can undergo various types of covalent modifications such as conjugation with carbonyl compounds such as 4-HNE and MDA elevated during lipid peroxidation under oxidative stress [54, 90, 131] or reactive metabolites of acetaminophen, produced during the metabolism of potentially toxic compounds [77–79, 124, 129]. In fact, the number of oxidatively modified proteins in acetaminophen-exposed liver tissues appears relatively small ([132], and Abdelmegeed et al., unpublished observation) despite increased nitroxidative stress [124]. These data likely reflect the fact that oxidation of Cys residues in many proteins in acetaminophen-exposed tissues could be suppressed because of their prior interactions with the reactive metabolite *N*-acetyl-*p*-benzoquinone imine and thus cannot be detected by redox proteomics approaches. However, these types of irreversible adduct formations of critical Cys residues of target proteins can be evaluated by the recovery of the functional activities after incubation with a strong reducing agent such as DTT. If the activities are restored by DTT, protein Cys residues could be modified through formation of reversible sulfenic acids or disulfides including mixed disulfides. If the activities are not recovered, Cys residues are likely modified through irreversible adducts formation [54, 90, 133] or hyperoxidation of Cys residues to sulfinic (−SOOH) and sulfonic (−SOOOH) acids ([17], and references herein). The possibility of these types of irreversible modification can be further confirmed by immunoprecipitation of the target protein followed by immunoblot analysis with anti-4-HNE or anti-acetaminophen antibody.

## 5. Applications of Redox Proteomics Approaches to Detect Oxidized Proteins in Other Subcellular Organelles, Many Other Tissues, and Different Disease States

We have thus far described oxidative modifications of mitochondrial proteins and their functional consequences in experimental animal models of fatty liver disease. However, it is quite logical to predict that proteins located in other subcellular organelles (e.g., cytoplasm, ER, and nuclear fractions) can also be oxidatively modified and thus contribute to tissue injury. For instance, oxidative inactivation of ER-resident chaperone proteins (e.g., protein disulfide isomerase and other heat shock proteins) can cause misfolding or unfolding of their client proteins, resulting in the unfolded protein response and ER stress. Oxidative modifications and potential inactivation of nuclear proteins such as DNA repair enzymes including O^6^-methylguanine-DNA-methyltransferase [117] or Ogg1 [56] could explain the increased levels of oxidatively modified DNA after exposure to potentially toxic compounds or under pathological conditions.

To understand the mechanism of ER stress and its pathological role, we also applied the simple biotin-switch redox proteomics method to systematically characterize oxidatively modified hepatic proteins in cytoplasm and ER from experimental animals of alcoholic and nonalcoholic fatty liver disease [134–136]. Our redox proteomics data showed that many ER-located chaperone proteins including protein disulfide isomerase, heat shock proteins, and other antioxidant enzymes including cytosolic SOD (SOD1) and peroxiredoxin are oxidized and inactivated [134, 135]. Consistent with these results, we observed increased unfolded protein responses in alcohol-exposed E47-HepG2 hepatoma cells and experimental animals (unpublished observations). It is also possible that these proteins can be inhibited through adduct formation with MDA or 4-HNE, as recently reported [133]. All these results suggest that increased ER stress, possibly originated from mitochondrial dysfunction [95] or uncoupled cytochrome P450 catalytic cycle [130], may also contribute to tissue damage.

Under specific pathological conditions or disease states, certain selected tissues are negatively affected. For instance, brain tissues are selectively distressed in neurodegenerative disorders [50] or after exposure to neurotoxic agents, while heart and blood vessels can be compromised in cardiovascular disorders. It is known that nitroxidative stress is a common factor in these pathological conditions in various tissues (Figure 1). However, it is poorly understood which proteins are oxidatively modified under different disease states. By analyzing oxidatively modified proteins in different tissues, we can also predict functional alterations of each target protein. In addition, it is of interest whether similar sets of mitochondrial proteins are oxidatively modified in different organs/tissues (e.g., liver versus other extrahepatic tissues such as brain, heart, lung, kidney, pancreas, and intestine) or different species (e.g., rodents versus humans) when analyzed by the redox proteomics approach. Our unpublished preliminary results indicate that the overall patterns of oxidized proteins in MDMA-exposed brain tissues are similar to those of the MDMA-exposed liver tissues, except for the liver-specific proteins including the enzymes involved in the mitochondrial fat oxidation pathway. By comparing the patterns of oxidative protein modifications in different tissues and species, we can estimate the role of specific proteins in mitochondrial dysfunction and disease progression of each organ or disease state.

## 6. Potential Translational Applications of Redox Proteomics Approaches to Evaluate Beneficial Agents to Prevent or Treat Mitochondrial Dysfunction

Once we understand the mechanism of mitochondrial dysfunction and ER stress, contributing to tissue injury, it is desirable to develop an effective strategy of prevention or therapy against mitochondrial dysfunction and organ damage based on our knowledge. We believe that the redox-based proteomics method can be used in translational research by evaluating the effectiveness or progress of treatment with a certain beneficial agent (e.g., antioxidants or cell protective agents from natural and synthetic origins). This task can be achieved by monitoring the levels of oxidatively modified mitochondrial proteins in the biological specimens before and after treatment with a beneficial agent. For instance, we have recently demonstrated a beneficial effect of a diet containing polyunsaturated fatty acids (PUFA) with physiological levels of arachidonic and docosahexaenoic acids on effectively preventing protein oxidation, mitochondrial dysfunction, and ultimately alcoholic fatty liver [137]. These results observed in rats are consistent with the beneficial effects of PUFA diets against alcoholic fatty liver in monkeys [138] and nonalcoholic fatty liver in rats fed a choline-deficient high fat diet [139]. Our results also provide the underlying mechanisms by which physiologically relevant levels of PUFA exert beneficial effects against alcoholic fatty liver in both rats [137] and monkeys [138].

**Figure 4:**
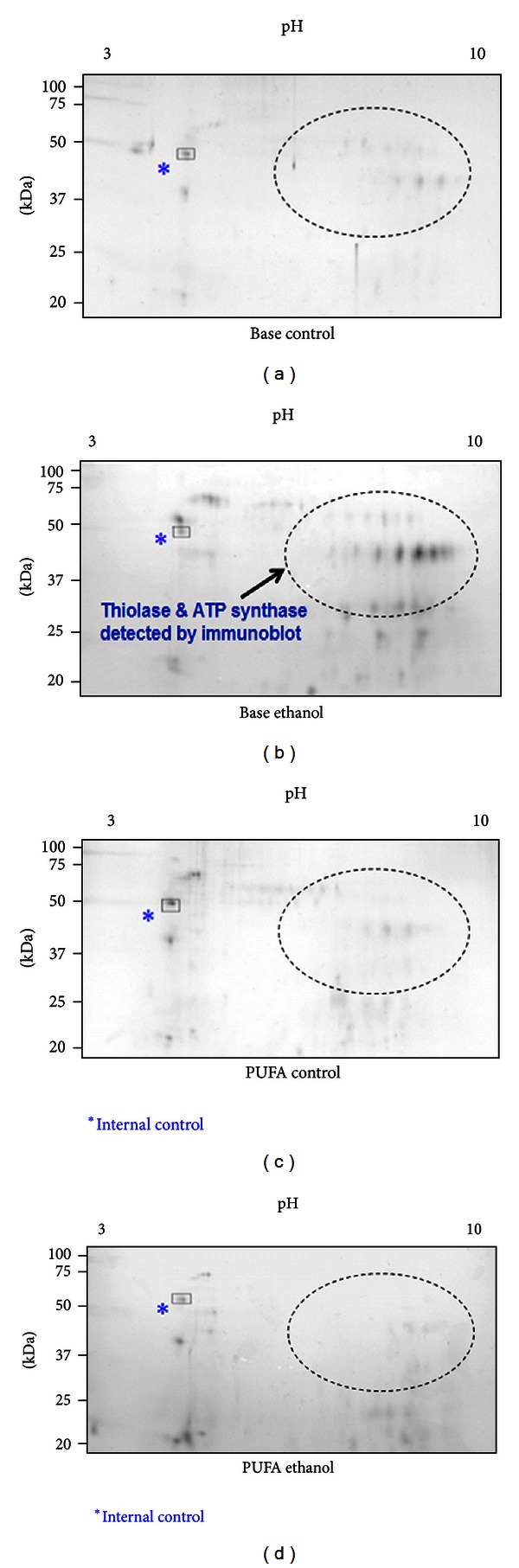
Translational research application of a redox proteomics approach by analyzing the oxidatively modified proteins in control and alcohol-exposed rats in the absence or presence of PUFA. Oxidatively modified mitochondrial proteins from each group were identified with a redox proteomics method using biotin-NM as a probe [106, 107], purified with streptavidin-agarose beads, then displayed on 2D gel, and stained with silver. The images of all gels were adjusted by optimizing the similar density of an endogenous, internal protein (*) in each gel. Protein spots in encircled areas in indicated samples reflect the appearance or disappearance of oxidized proteins depending on the treatment in each group, as described in and adapted from [137].

As shown in Figure 4, the number and levels of oxidatively modified mitochondrial proteins were increased in alcohol-fed control rats (Base ethanol) compared to pair-fed control rats (Base control). Our results [137] showed that increased production of hydrogen peroxide and peroxynitrite in alcohol-exposed rats (Base ethanol) compared to pair-fed control group (Base control). These results are consistent with elevated levels of CYP2E1 and iNOS in ethanol-fed rats. Immunoblot analyses of oxidized proteins from each group revealed the presence of oxidatively modified thiolase and *α*-ATP synthase only in the Base-ethanol group. However, the increased levels of oxidized proteins in the Base-ethanol group were markedly decreased in rats fed the same amounts of alcohol in the presence of PUFA (PUFA-ethanol). Addition of PUFA to ethanol-fed rats (PUFA-ethanol) improved histological data (i.e., disappearance of fat vacuoles) with the absence of the oxidized protein bands of both thiolase and *α*-ATP synthase detected only in the Base-ethanol group. Furthermore, the respective activities of thiolase, ATP synthase, and ALDH2, all suppressed in the Base-ethanol group, were restored in the PUFA-ethanol group. Further mechanistic studies revealed that the PUFA diet significantly prevented activation/induction of CYP2E1 and iNOS, which produce ROS and RNS, respectively, observed in alcohol-exposed tissues (Base ethanol). Consequently, the elevated levels of a potently toxic peroxynitrite, which can *S*-nitrosylate Cys residues and/or nitrate Tyr residues of various proteins [48], were significantly decreased in the PUFA-ethanol group compared to those in alcohol-fed control rats (Base ethanol).

During our mechanistic study on I/R-related mitochondrial dysfunction, we were also able to evaluate the beneficial effect of a peroxynitrite scavenger metalloporphyrin MnTMPyP against mitochondrial dysfunction and acute hepatic I/R injury [70]. MnTMPyP pretreatment markedly suppressed the I/R-related elevation of serum transaminase levels, histological damage, iNOS expression, and oxidative modifications of key mitochondrial proteins assessed by comparative 2D gel analysis for each sample. These changes were further supported by the activity measurements of mitochondrial ALDH2, thiolase, and ATP synthase as well as histopathological evaluation. These studies provide supporting evidence for a translational research application of the redox proteomics approach against mitochondrial dysfunction and organ damage in numerous disease states. We expect that beneficial effects of many natural antioxidant agents such as polyenephosphatidylcholine [140, 141], *S*-adenosylmethionine [142], resveratrol [143], curcumin [144, 145], and silymarin [144, 145] or various synthetic agents such as vitamin E analogs and carvedilol [146] against mitochondrial dysfunction and oxidative tissue injury could be demonstrated by using the redox-based proteomics approaches. Furthermore, the redox proteomics approach may also be used in finding potential biomarkers of disease states in other extrahepatic tissues including brain, heart, lung, and kidney from experimental models as well as human tissue specimens.

## Abbreviations

ALDH2: Mitochondrial low-Km aldehyde dehydrogenase 2
BIAM: Biotin-conjugated iodoacetamide
Biotin-NM: Biotin-*N*-maleimide
Complex I: NADH-dependent ubiquinone oxidoreductase
Complex III: Cytochrome c reductase
Complex IV: Cytochrome c oxidase
CYP2E1: Ethanol-inducible cytochrome P450 2E1 isozyme
DIGE: Difference in gel electrophoresis
ER: Endoplasmic reticulum
ETC: Electron transport chain
4-HNE: 4-Hydroxynonenal
iNOS: Inducible nitric oxide synthase
I/R: Ischemia-reperfusion
MDA: Malondialdehyde
MDMA: 3,4-methylenedioxymethamphetamine
NEM: *N*-ethylmaleimide
NO: Nitric oxide
PUFA: Polyunsaturated fatty acids
RNS: Reactive nitrogen species
ROS: Reactive oxygen species
*S*-NO-Cys: *S*-nitrosylated Cys
SOD: Superoxide dismutase
Thiolase: 3-Ketoacyl-CoA thiolase.
